# An in vitro workflow of neuron-laden agarose-laminin hydrogel for studying small molecule-induced amyloidogenic condition

**DOI:** 10.1371/journal.pone.0273458

**Published:** 2022-08-26

**Authors:** Poommaree Namchaiw, Patapon Bunreangsri, Piyaporn Eiamcharoen, Salita Eiamboonsert, Rungtiva P. Poo-arporn

**Affiliations:** 1 Biological Engineering Program, Faculty of Engineering, King Mongkut’s University of Technology Thonburi, Thung Kru, Bangkok, Thailand; 2 Neuroscience Center for Research and Innovation, Learning Institute, King Mongkut’s University of Technology Thonburi, Thung Kru, Bangkok, Thailand; 3 Department of Pathology, Faculty of Veterinary Medicine, Kasetsart University, Thung Kru, Bangkok, Thailand; 4 Veterinary Medical Teaching Hospital, University of California Davis, Davis, California, United States of America; 5 Media Technology, King Mongkut’s University of Technology Thonburi, Thung Kru, Bangkok, Thailand; McGill University, CANADA

## Abstract

In vitro studies have been popularly used to determine the cellular and molecular mechanisms for many decades. However, the traditional two-dimension (2D) cell culture which grows cells on a flat surface does not fully recapitulate the pathological phenotypes. Alternatively, the three-dimension (3D) cell culture provides cell-cell and cell-ECM interaction that better mimics tissue-like structure. Thus, it has gained increasing attention recently. Yet, the expenses, time-consuming, and complications of cellular and biomolecular analysis are still major limitations of 3D culture. Herein, we describe a cost-effective and simplified workflow of the 3D neuronal cell-laden agarose-laminin preparation and the isolation of cells, RNAs, and proteins from the scaffold. To study the effects of the amyloidogenic condition in neurons, we utilized a neuron-like cell line, SH-SY5Y, and induced the amyloidogenic condition by using an amyloid forty-two inducer (Aftin-4). The effectiveness of RNAs, proteins and cells isolation from 3D scaffold enables us to investigate the cellular and molecular mechanisms underlying amyloidogenic cascade in neuronal cells. The results show that SH-SY5Y cultured in agarose-laminin scaffold differentiated to a mature TUJ1-expressing neuron cell on day 7. Furthermore, the gene expression profile from the Aftin-4-induced amyloidogenic condition revealed the expression of relevant gene-encoding proteins in the amyloidogenic pathway, including *APP*, *BACE1*, *PS1*, and *PS2*. This platform could induce the amyloid-beta 42 secretion and entrap secreted proteins in the scaffold. The induction of amyloidogenic conditions in a 3D culture facilitates the interaction between secreted amyloid-beta and neurons, which makes it resembles the pathological environment in Alzheimer’s brain. Together, this workflow is applicable for studying the cellular and molecular analysis of amyloid-induced neuronal toxicity, such as those occurred in Alzheimer’s disease progression. Importantly, our method is cost-effective, reproducible, and easy to manipulate.

## Introduction

Amyloidogenesis is the abnormal aggregation of amyloid peptides. It involves complications in several neurological diseases and disorders such as Alzheimer’s disease, Parkinson’s disease, Down’s syndrome, fragile X, epilepsy, and autism [1]. Specifically, Alzheimer’s disease is the common cause of dementia in the elderly worldwide. The pathological hallmarks of the disease are amyloid plaques and neurofibril tangles, which are predominantly present in an Alzheimer’s brain but slightly present in a healthy elderly’s brain [2]. In general, amyloid peptides are the byproduct from a proteolysis of amyloid precursor proteins (APP). The cleavage of amyloid precursor proteins, either by α-secretase or β-secretase, leads to the secretion of amyloid precursor protein ectodomains, called sAPPα and sAPPβ, respectively. The sAPPα domains are subsequently cleaved by γ-secretase to generate the p3 domain, while sAPPβ cleavage generate amyloid-β. Only the amyloid precursor proteins processing through β-secretase and γ-secretase is included in the amyloidogenic pathway, generating amyloid-β polypeptide of 32–47 amino acids [1, 3]. The amyloid-β 40 is the most abundant isoform, while the amyloid-β 42 is an aggregation-prone isoform [4, 5], in which the latter predominantly presents in the amyloid plaque of an Alzheimer’s brain [6]. The aggregation of amyloid-β 42 correlates with the disease severity [7]. However, the dysregulation of APP catabolism that favors the build-up of amyloid-β 42 is still unknown. Familial Alzheimer’s disease (FAD) may result from the genetic mutation of genes in the amyloidogenic pathway, such as, amyloid precursor protein; *APP* and presenilin subunits in the γ-secretase complex; *PS1* and *PS2* [8]. However, most of the cases do not inherit these mutations, so called sporadic Alzheimer’s disease (SAD) which occurs at the late-onset or over 65-year-old.

The increase of aging society might have led to the increase in the prevalence of Alzheimer’s cases worldwide. The prevention and curation of the disease are thus needed. There are several existing two- dimension (2D) models for studying Alzheimer’s disease by using patient-derived induced pluripotent stem cells (iPSC) [9, 10]. Although it could replicate multiple pathological characteristics of the disease, the traditional 2D culture fails to induce the amyloid-β accumulation. Furthermore, they also lack cell-to-cell and cell-to-extracellular matrix (ECM) interaction. In attempts to mimic the cellular microenvironment, cerebral organoid or mini-brain cultures were developed. The neural progenitors are differentiated from human iPSC as embryoid bodies. This allows cells to grow in three-dimension (3D) and increases cell-to-cell interaction [11]. However, the secreted amyloid-β in the 2D cell culture and cerebral organoid system is often removed from cell cultures during routine medium change, which lead to a lower chance of neural cells exposed to the neural toxic peptides. It is, therefore, critical to have a simplified method that captures the pathological hallmarks of the disease. The cell culture in matrices scaffolds such as natural polymer, synthetic polymer, porous scaffold, and fibrous scaffold shed the light in Alzheimer’s disease in vitro model [11, 12]. 3D culture in scaffold matrices allows the secreted protein to be entrapped inside the scaffolds, and thus facilitates the exposure of cells to secreted amyloid-β. Despite the 3D culture in scaffolds enhances pathological appearances similar to spontaneous disease pathogenesis, the isolation of cells and biomolecules such as DNA, RNA, and protein from scaffold matrices remains a challenge [13, 14].

Previous studies used patient-derived iPSC carrying *APP* mutations such as *APPswe*, *APPV717I*, *PS1*, and *PS2* mutation to generate the Alzheimer’s model in vitro [15–17]. In spite of the use of FAD-iPSC, small molecules, called Aftin-4, have been found to induce the secretion of amyloid-β 42 in several platforms including mouse models, ex vivo, and in vitro [18–20]. This could replicate several pathological characteristics such as the amyloid-β 42 accumulation, cellular prion protein (PrP^C^) expression, and mitochondrial impairment [19–21]. Although the 3D culture of FAD-iPSC has been popularly used recently, the time consumption, expense, complexity, and reproducibility are still the main difficulties and drawbacks of such models [22, 23]. The difficulties to utilize iPSC in test models are the heterogenicity of neural cell types derived from human iPSC, as well as long induction time to differentiate human iPSC into neuronal cells. Thus, several researchers have been using immortalized neural progenitor cells (ReN cell), mouse neural crest-derived cell line (N2a), and human neuroblastoma (SH-SY5Y), either with or without FAD-related genes to generate Alzheimer’s model in a culture dish [19, 20, 24].

A human neuroblastoma cell line; SH-SY5Y can differentiate towards neuron-like cells expressing beta tubulin-III (TUJ1+) and several neuronal-specific markers including dopaminergic neurons (TH, DAT, D2R, D3R), cholinergic neurons (ACHE, nAChR), and adrenergic neuron (NET, VMAT) [25, 26]. Those cells are involved in the impairment of several neurodegenerative disorders, such as Parkinson’s and Alzheimer’s diseases. In this study, we implement the use of SH-SY5Y cell line in agarose-laminin gel-based 3D culture system. The culture of SH-SY5Y cells in agarose-laminin gel-based is simple and SH-SY5Y cells can be differentiated to neuron-like cells by the supplementation of retinoic acid (RA) and brain-derived neurotrophic factor (BDNF) [26–28]. In this study, we successfully induced the secretion of amyloid-β 42 by using the small molecule Aftin-4. This model had been shown to entrap amyloid-β 42 in the scaffolds and increase the amyloid-β 42/40 ratio, which is one of the pathological characteristics in both FAD and SAD [29–31]. This method was a simple and affordable approach and required about one week of the experiment. The cells derived from the 3D scaffold could be retrieved by disassembling the agarose structure with microwave irradiation. The RNA and protein of the cell-laden agarose-laminin scaffolds could be isolated by using the well-established TRIzol extraction method with some modifications. The derived RNA, protein, and cell from the 3D scaffolds enabled us to identify the molecular and cellular responses under amyloidogenic conditions. In addition, the use of small molecules to induce amyloidogenic conditions instead of viral plasmid infection is more convenient and easier to manipulate in most biosafety level laboratories.

## Materials and methods

### Routine maintenance of SH-SY5Y cell line

SH-SY5Y, originally purchased from American Type Culture Collection (ATCC, Rockville, MD, USA), was kindly provided by Dr. Y. Jaisin (Srinakarinwirot University, Thailand). The cell line was expanded as an adherent cell culture (2D) in a basal medium supplemented with 10% fetal bovine serum (FBS) (cat. No. 10437–010, Gibco, Massachusetts, USA). Cells were sub-cultured when reaching 70% confluence by using Trypsin-EDTA, 0.25% (cat. No. 25200056, Gibco, Massachusetts, USA). Cells were then centrifuged at 500 x g for 5 minutes at room temperature and seeded at 1:6 ratio on a tissue culture treated plate under maintenance culture medium.

### Cell-laden agarose-laminin preparation

SH-SY5Y cells were dissociated with Trypsin-EDTA, 0.25% (cat. No. 25200056, Gibco, Massachusetts, USA) for 5 minutes at 37°C, then centrifuged at 500 x g for 5 minutes at room temperature, prior to resuspended with basal medium supplemented with 10% FBS (cat. No. 10437–010, Gibco, Massachusetts, USA) and maintained at 37°C at the desired density. The SeaPlaque low-melting point agarose (cat. No. A1315, PhytoTechnology, Kansus, USA) were prepared at the concentration of 3% agarose with basal medium. Cell suspension, pre-warmed basal medium and 3%agarose were mixed at 2:1:1 ratio to get the seeding density of 2x10^6^ cells/mL with final concentration of 0.75% agarose and 5% FBS. The cell-gel suspension was then added with 5 μg/mL laminin (cat. No. L-2020, Sigma-Aldrich, MO, USA) and then plated on the 24-wells tissue culture plate (50 μL per well). Agarose gel was allowed to form a rapid gelation at room temperature. Cell-laden agarose-laminin scaffolds were then cultured under 500 μL of basal medium containing 5% FBS and incubated at 37°C, 5% CO_2_ and 95% humidity (noted as day 0; DIV0).

### Differentiation of SH-SY5Y in agarose-laminin scaffolds

On day 1, half of cell culture medium were replaced with fresh basal medium to get the final FBS concentration of 2.5% serum. The following day (day 2), the entire culture medium was replaced with the differentiation medium (basal medium containing 1 μM retinoic acid (cat. No. R2625 Sigma, St. Louis, USA) and 10 ng/mL BDNF (cat. No.450-02, Peprotech, New Jersey, USA)). Cells were replaced with the differentiation medium every other day.

### Small molecule-induced amyloidogenic condition in vitro

The stocking Aftin-4 solution (cat. No. SML-1578, Sigma-Aldrich, MO, USA) was freshly prepared at 500X concentration in DMSO. On day 6, the cell-laden agarose-laminin scaffolds were treated with Aftin-4 for 24 hours at the final concentration of 0, 25, and 50 μM (vehicle control, 0.2% DMSO) by direct adding 1 μL of Aftin-4 solution into the scaffolds, which submerged in 500 μL cell culture medium. The scaffolds were collected for further analysis on day 7.

### Total RNA extraction using TRIzol extraction method

The total RNA was extracted with TRIzol reagent following manufacturer’s recommendation with some modifications. In details, cell-laden agarose-laminin scaffolds were incubated with 300 μL TRIzol reagent (cat. No. 15596026, Invitrogen, Massachusetts, USA) per 50 μL of scaffold for 2 to 3 minutes at room temperature. The tube was then inverted several times until the scaffolds were completely melted. The homogenization was accomplished by adding 0.2 volume of chloroform (cat. No. AA32614K2, Fisher Scientific, Massachusetts, USA) and subsequently shaking vigorously for 15 seconds. The mixture was centrifuged at 12,000 x g for 15 minutes at 4°C. The upper aqueous phase was transferred to a new tube with a subsequent addition of 0.5 volume of isopropanol (cat. No. A416, Fisher Scientific, Massachusetts, USA) and then incubated at 4°C for 10 minutes. The homogenate was then centrifuged at 12,000 x g for 10 minutes at 4°C. The supernatant was then discarded. The RNA pellet was washed with 1 mL of 75% ethanol (cat. No. BP2818500, Fisher Scientific, Massachusetts, USA) and centrifuged at 7,500 x g for 5 minutes at 4°C. Then, the RNA pellet was resuspended in 50 μL UltraPure^TM^ DNase/RNase-free distilled water (cat. No. 10977015, Invitrogen, Massachusetts, USA) and heated to 60°C for 10 to 15 minutes to solubilize the RNA. At this point, the RNA may become gelation at the room temperature. The isolated RNA was then proceeded the agarose removal by using Zymoclean™ Gel RNA Recovery Kit following manufacture’s recommendation (cat. No. R1011, Zymo research, California, USA). The extracted RNA was eluted with 15 μL DNase/RNase-Free Water and stored at -80°C for further analysis. The total RNA concentration and RNA degradation were determined by Qubit RNA high sensitivity assay kit and Qubit RNA IQ assay kit (cat No. Q32855 and Q33221, Thermo Fisher, Massachusetts, USA). The data were acquired and analyzed on Qubit 4 Fluorometer. The RNA sizing and quality was determined by capillary electrophoresis (Bioanalyzer 2100, Agilent Technologies, Waldbronn, Germany).

### cDNA synthesis and qRT-PCR

The isolated RNA was further used for first-strand cDNA synthesis using iScript™ cDNA Synthesis Kit following manufacture’s recommendation (cat. No. 1708891, Bio-Rad, California, USA). The real-time RT-PCR was analyzed by using iTaq Universal SYBR Green Supermix (cat. No. 1725121, Bio-Rad, California, USA). The primers pairs used in this study were listed in Table 1 [32]. Real-time qRT-PCR was analyzed by CFX96 Real-time PCR detection system (Bio-Rad, California, USA).

**Table 1:** The list of primers used in this study.

Gene	Forward 5’>3’	Reverse 5’>3’	Primers bank ID [32]
***GAPDH***	ACAACTTTGGTATCGTGGAAGG	GCCATCACGCCACAGTTTC	378404907c2
***APP***	CAAGCAGTGCAAGACCCATC	AGAAGGGCATCACTTACAAACTC	228008405c1
***PS1***	GACGACCCCAGGGTAACTC	ACTGACTTAATGGTAGCCACGA	195947402c1
***PS2***	AGTGTGTGATGAGCGGACG	ACTGGGCAGTGTTCTCTCCAT	156105680c1
***BACE1***	TCTGTCGGAGGGAGCATGAT	GCAAACGAAGGTTGGTGGT	333440462c1
***TUJ1***	GGCCAAGGGTCACTACACG	CAGTCGCAGTTTTCACACTC	308235961c1

### Total protein extraction and analysis

The lower organic phase following TRIzol/chloroform extraction was transferred to a new tube and added with 0.3 volume of ethanol (cat. No. BP2818500, Fisher Scientific, Massachusetts, USA). The solution was mixed vigorously. The mixture was then added with 1.5 volume of isopropanol (cat. No. A416, Fisher Scientific, Massachusetts, USA) and incubated at room temperature for 10 minutes. The mixture was centrifuged at 12,000 x g for 10 minutes at 4°C and the supernatant was discarded. The protein pellet was washed with 1 mL 95% ethanol (cat. No. BP2818500, Fisher Scientific, Massachusetts, USA) and centrifuged at 7,500 x g for 5 minutes at 4°C. The protein pellet was then resuspended in 0.5% SDS, 1mM PMSF, 1mM Na-Orthovanadate in Ultra-Pure water. The total protein concentrations were determined by Micro BCA assay kit (cat. No.23235, Thermo Scientific, Massachusetts, USA). The human amyloid-β1–40 (cat. No. DAB140, R&D systems, Minnesota, USA) and amyloid-β1–42 (cat. No. DAB142, R&D systems, Minnesota, USA) were evaluated by Quantikine ELISA kits following manufacturer’s recommendation. The dot blot analysis was performed with 1 μg total protein. Briefly, the protein was dotted on nitrocellulose membrane and allowed to air dry. The membrane was blocked with OneStep blocker (cat No. BS001-B500mL, Bio-Helix, Massachusetts, USA), probed with actin-β at final concentration of 1:1000 (cat No. ABP50593, Abbkine, Wuhan, China) and secondary goat anti-rabbit IgG AF488 conjugated at final concentration of 1:250 (cat No. E-AB-1055, Elabscience, Wuhan, China). The membrane was washed 3 times with PBST buffer and analyzed with Bio-Rad ChemiDoc imaging system. In other experiments, the membrane was probed with amyloid-β-N biotinylated at final concentration of 1:5000 (cat No. 3740-6-250, MabTech, Sweden) and secondary streptavidin/HRP (kindly gifted by Dr. D. Waraho; King Mongkut’s University of Technology) at the final concentration of 1:2000. The membrane was washed 3 times with PBST buffer and probed with UltraScence Pico Plus Western Substrate (cat No. CCH321-B100mL, Bo-Helix, Massachusetts, USA). The membrane was then analyzed with Bio-Rad ChemiDoc imaging system.

### Cell retrieval from agarose-laminin scaffolds

Gel was fixed with 4% PFA for 15 minutes at room temperature. One to three gels were collected in 1.5 mL tube filled with 500 μL PBS buffer. Cells were then performed intermittent microwave irradiation 5 cycles of 4 seconds on and 4 seconds off. Cells was immediately collected by spinning down at 500 x g for 5 minutes at room temperature. The retrieved cells were then dissociated to a single cell suspension by using 300 μL Trypsin-EDTA, 0.25% (cat. No. 25200056, Gibco, Massachusetts, USA) for 5 minutes at 37°C. The cells were collected by centrifugation at 500 x g for 5 minutes and resuspended in PBS containing 10% FBS in PBS prior to proceeding immune staining and flow cytometry analysis.

### Cell counting

To evaluate the recovery percentage from cell retrieval method, we prepared cell-laden agarose-laminin scaffold at the seeding density of 1x10^5^ cells per scaffold. After allowing them to became a gelation stage, the cells were retrieved from scaffold as mentioned above. We used Muse count & viability kit (Luminex Corporation, Texas, USA) to determine the cell counts and viability. In brief, the retrieved cells were resuspended in 50 μL PBS buffer. Cell suspension was then mixed with 450 μL Muse® Count & Viability Reagent and incubated at room temperature for 5 minutes. The results were acquired by Guava® Muse® Cell Analyzer.

### Immunostaining for flow cytometry

The retrieved cells were resuspended in ice cold 10% FBS in PBS buffer. The cell suspension was incubated with primary antibody; mouse mAb anti-Nestin (1:500; cat. no. MAB5356, Millipore, Massachusetts, USA), or mouseIgG2a anti-Tubulin β 3 (1:500; cat. no.801202, Biolegend, San Diego, USA) for 15 minutes on ice and followed by secondary antibody goat anti-mouse IgG Alexa Fluor^TM^ 488 (Invitrogen, cat. no. A-11001, Massachusetts, USA) staining. Cells were then collected by spun down at 500 x g for 5 minutes and resuspended in 10% FBS in PBS buffer. The flow cytometry results were acquired by BD FACSCalibur^TM^ and analyzed by FlowJo V.10 software.

### Statistical analysis

All data were present as mean ± standard error. The statistical analysis of amyloid-β and q-RT PCR were analyzed using one-way analysis of variance (one-way ANOVA) followed by Dunnett’s post-hoc analysis. The significant difference between mean was indicated at the level of *P-value* < 0.05.

### Cell culture media

#### Basal medium

Dulbecco’s modified Eagle’s medium/Nutrient mixture F-12 (DMEM/F12, cat. No.12500062, Gibco, Massachusetts, USA), 1x penicillin/ streptomycin (cat. No. 15140–122, Gibco, Massachusetts, USA) and 15mM HEPES (cat. No.H2393, SigmaAldrich, St. Louis, USA).

#### Maintenance cell culture medium

Basal medium supplemented with 10% FBS (cat. No. 10437–010, Gibco, Massachusetts, USA).

#### Differentiation medium

Basal medium supplemented with 1μM retinoic acid (cat. No. R2625 Sigma, St. Louis, USA) and 10 ng/mL BDNF (cat. No.450-02, Peprotech, New Jersey, USA).

## Results

### Preparation of cell-laden agarose-laminin scaffold

In the past few years, a 3D culture system has become more popular over the conventional 2D system because they could better represent an actual environment in vivo and provide a higher predictivity in pathological characteristics. The 3D culture can be grown with or without the presence of a scaffold [33]. When cultured without scaffold, neurons are cultured based on spheroid formation, for example, hanging drop, free-floating, and bioprinting systems [34]. Among these, a free-floating (also known as “scaffold-free”) method is the most widely used system. This method has been used for decades, especially to generate neurosphere. The free-floating system is comparatively easy to manipulate than others. However, the maintenance of the spheroid size, the ratio of cell maturation, and nutrient distribution are practical challenges for free-floating spheroid culture. In contrast, scaffold 3D structure overcomes these limitations of the free-floating system as it could manipulate the porosity, the permeability and the cell seeding density [35, 36]. In this workflow, we demonstrated the generation of a cell-laden agarose-laminin scaffold for inducing amyloidogenic conditions in vitro.

**Fig 1:**
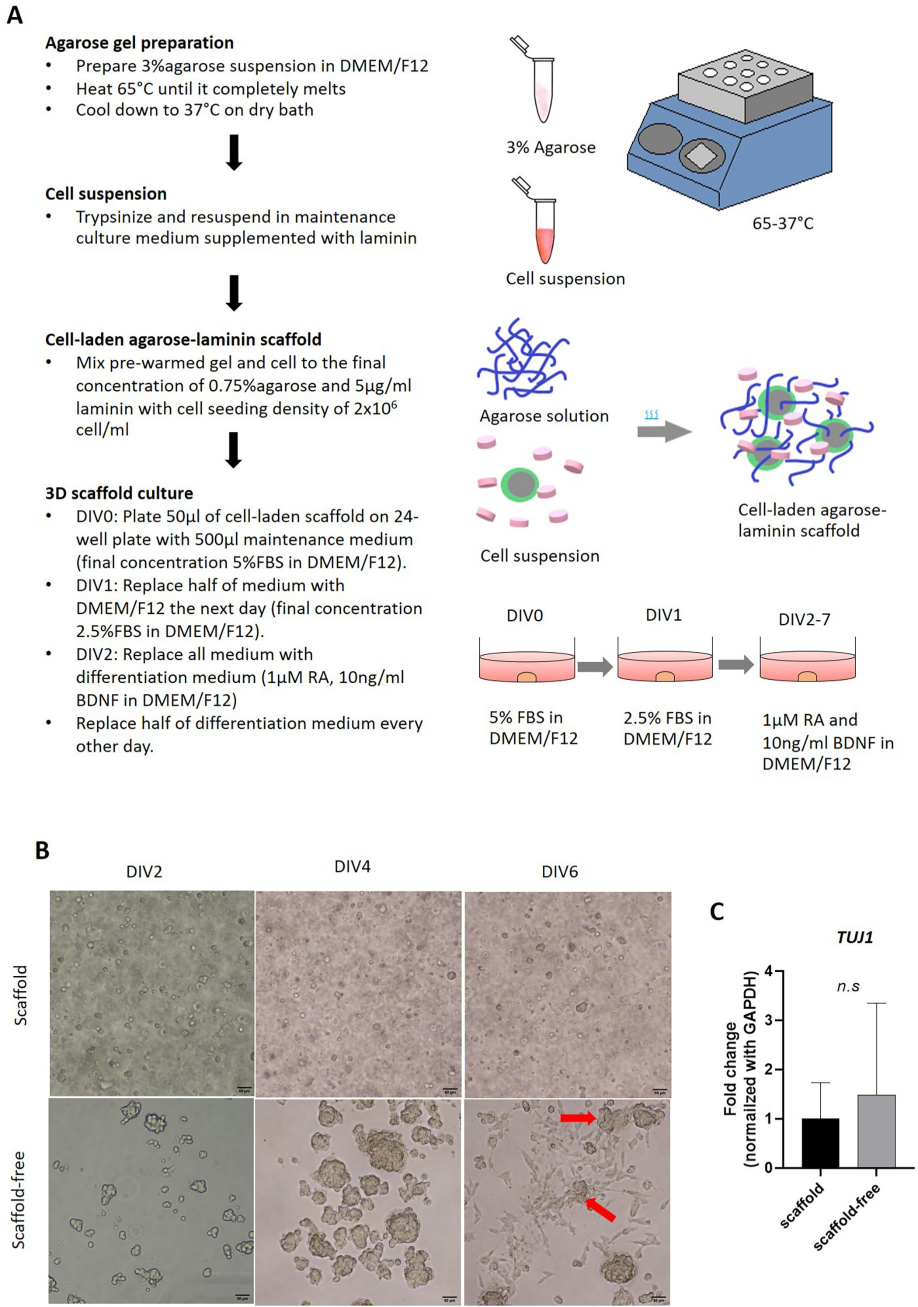
A neuron-laden agarose-laminin hydrogel. A) A workflow diagram for cell-laden agarose-laminin scaffold preparation B) The morphology of 3D culture in two different platforms; scaffold (top) and scaffold-free (bottom) culture at day 2, 4, and 6 post-seeding. At day 6 of cell culture under scaffold-free platform, SH-SY5Y cells were aggregated and began to attach on the plastic surface (red arrow). Scale bar represents 50 μm. C) The expression of mature neural marker, *TUJ1* of scaffold and scaffold-free 3D cultures at day 7. Bar graph represents the mean ± SE (n = 3). n.s: not statistically different.

The general timeframe of the cell-laden agarose-laminin scaffold culture system was ranged from 15 minutes to 30 minutes and required a temperature-controlled dry bath. We prepared working agarose solution at 3% w/v in a maintenance cell culture medium. The agarose was melted at 65°C and cooled down to 37°C in a dry bath. The SH-SY5Y cells were dissociated to single-cell suspension using 0.25% Trypsin-EDTA. Then, the cells were spun down at 500 x g for 5 minutes at room temperature and the supernatant was discarded. The cell pellet was resuspended in a maintenance cell culture medium containing FBS to the desired seeding density and incubated at 37°C until agarose was cooled down from 65°C to 37°C. Once the agarose was ready, laminin and pre-warmed agarose were added into the cell suspension to get a final concentration of 5% FBS, 0.75% agarose, and 5 mg/mL laminin with 2x10^6^ cells/mL seeding density. The cell-gel mixture was plated on a 24-well cell culture plate as a single droplet of 50 μL (1x10^5^ cells/scaffold). The cell-laden scaffold was allowed to form rapid gelation at room temperature. The 3D scaffold was then cultured with maintenance cell culture medium containing 5% FBS overnight then reduced to 2.5% FBS serum depleted cell culture medium the following day. After that, the cell culture medium was switched to the differentiation medium and was replaced every other day (Fig 1A).

In parallel, we also seeded 1x10^5^ cells on an untreated 24-well plate to generate a 3D scaffold-free culture counterpart. The results showed that cells were capable to proliferate in the agarose-laminin scaffold. The diameter of the spheroids increased approximately 1.7 times the single-cell size after 2 days in a maintenance cell culture medium. Moreover, the size of the spheroids did not change once cultured in a differentiation medium. Cell-laden agarose-laminin scaffolds gave rise to mostly uniform spheroids in size in comparison with their scaffold-free counterpart, which had a high variation of spheroid size and became enlarged even in the differentiation medium. In addition, we observed the aggregation of spheroids in scaffold-free culture, which later attached on a plastic surface (Fig 1B). In addition to the size distribution, we evaluated the differentiation of neuron-like cell line by measuring the expression of the maturation marker; *TUJ1*. We found that cells derived from DIV7 of cell-laden agarose-laminin scaffolds had a higher *TUJ1* expression (1±0.74 folds) than cells derived from scaffold-free cultures (1.48±1.86 folds), but this was not statistically different (*P-value* = 0.82, n = 3) (Fig 1C). An expression of *TUJ1* of cells in scaffold-free culture also had a higher variation than cells derived from the scaffold system. This result may relate to the high variation of spheroid size, which affected the nutrient distribution of each spheroid.

### Induction of amyloid-β deposit in an agarose-laminin scaffold and isolation of proteins from the agarose-laminin scaffold

**Fig 2:**
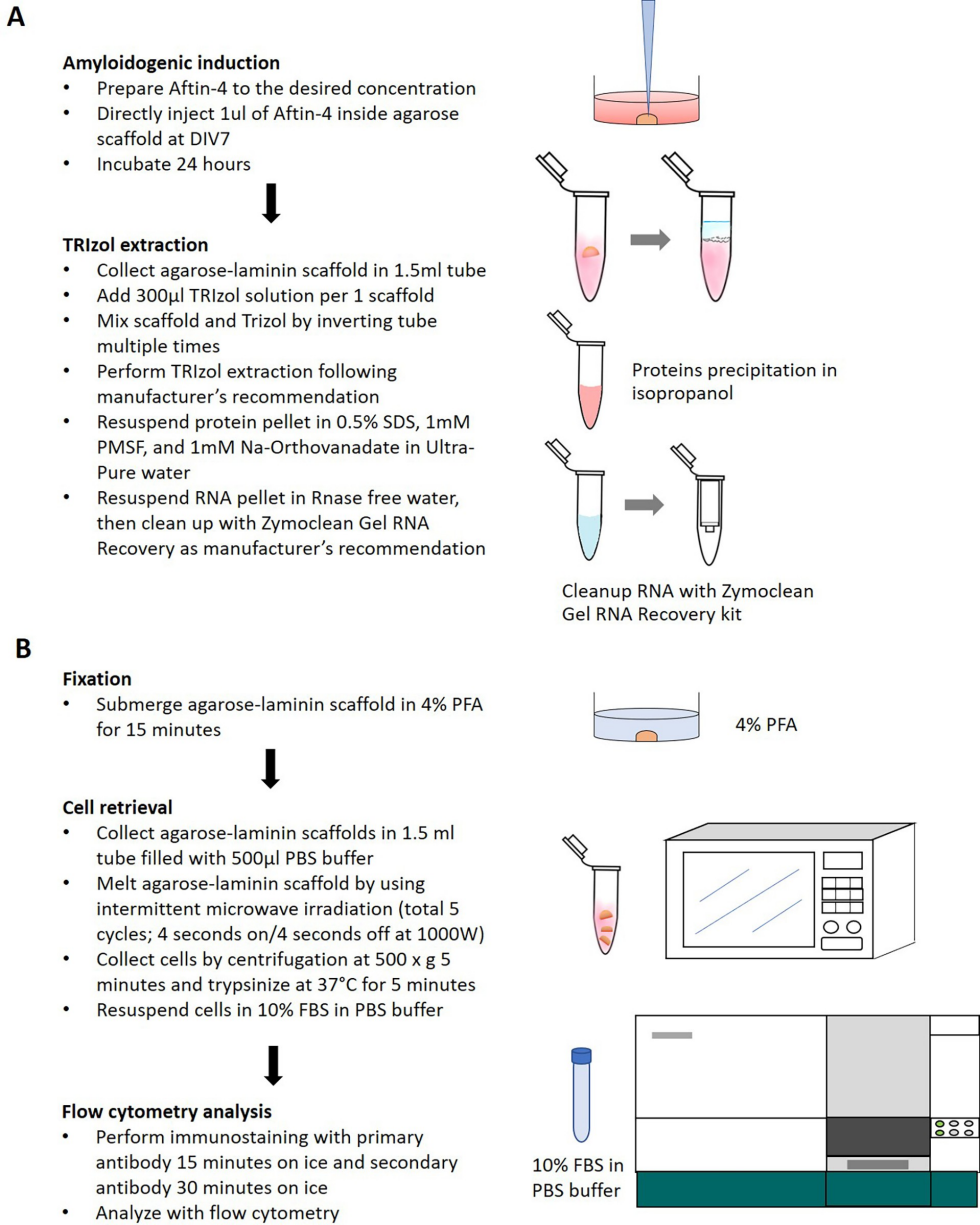
An amyloidogenic induction in vitro A) A workflow diagram of the amyloidogenic induction in cell-laden agarose-laminin scaffold. B) A workflow diagram of the cell retrieval from cell-laden agarose-laminin scaffold.

**Fig 3:**
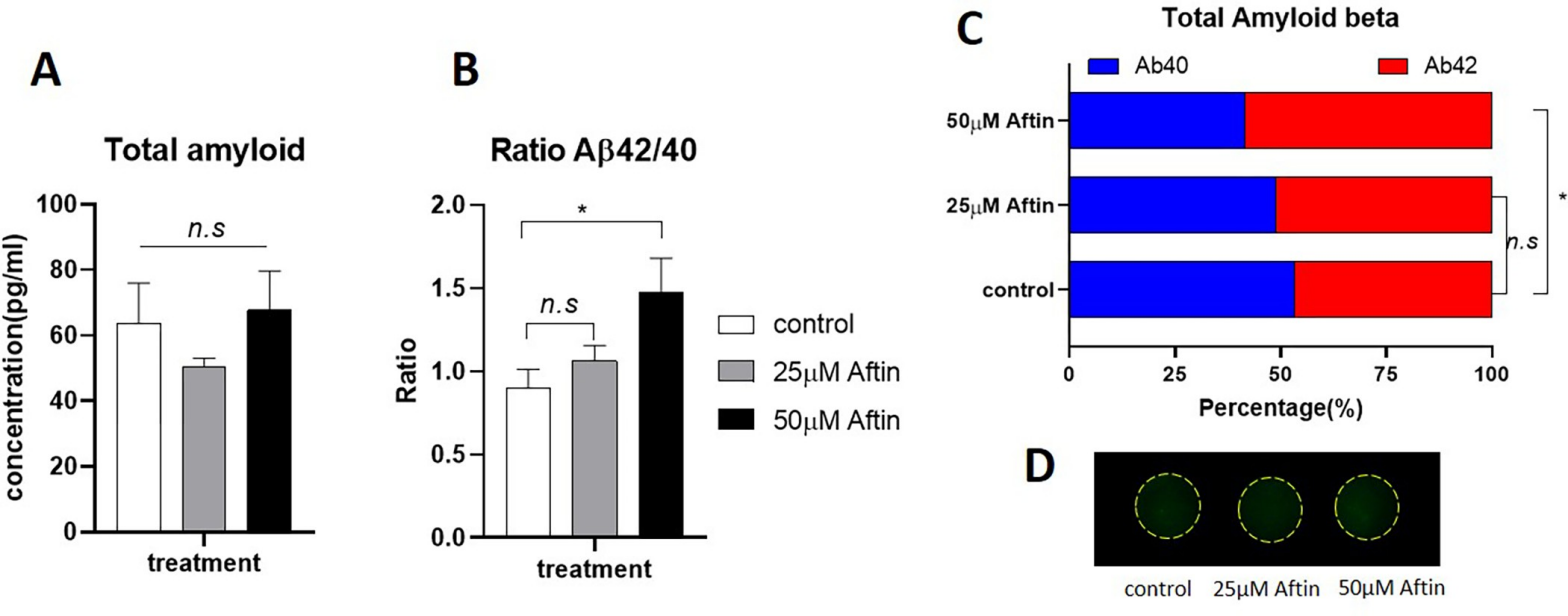
Small molecule-induced amyloid-β 42 production A) The concentration of total amyloid-β (40+42 isoforms) analyzed by ELISA assay. B) The ratio of total amyloid-β 42 to amyloid-β 40. C) The percentage of two isoforms in total amyloid-β. (n = 5) *: p<0.05. n.s: not statistically different. D) A total protein of 1 μg was blotted on nitrocellulose membrane and probed with actin-β antibody.

Amyloid-β plaque is one of the characteristic features of Alzheimer’s disease. The hypothesis of amyloid-β accumulation, called “amyloid cascade hypothesis” is believed to be the initial step in Alzheimer’s, which leads to other pathological symptoms [6, 37]. To induce the production of amyloid-β 42 in cell culture, we treated cells with Aftin-4, an amyloid forty-two inducer small molecule which modulate the β- and ɣ-secretase activity [19]. Cells were treated with Aftin-4 at the sub-lethal dose of 25 μM and the lethal dose of 50 μM for 24 hours (Aftin-4 IC50 = 43.88 μM, determined by 2D cell culture). To evaluate the small molecule-induced amyloidogenic condition in 3D culture, we isolated proteins from the agarose-laminin scaffold by using conventional TRIzol extraction method (Fig 2A). We could isolate approximately 8 to 15 μg total protein from 1x10^5^ cells. The total protein of 1 μg was used to determine amyloid-β concentration using Quantikine ELISA kit DAB140B and DAB142. The results showed that total amyloid-β (Aβ-40 and Aβ-42) were consistent among 3 conditions; control, 25 μM Aftin-4, and 50 μM Aftin-4 during 24 hours of treatment (Fig 3A). The amount of total amyloid production was higher when cells treated with 50 μM Aftin-4 (67.77±26.76 pg/mL), comparing to the treatment with 25 μM Aftin-4 (50.36 ± 5.95 pg/mL). However, this difference was not statistically significant among conditions (*P-value* = 0.42) when compared to the vehicle control group (63.80 ± 24.32 pg/mL). However, the alteration of amyloid beta-42/40 ratio was increased from 0.90 in the controls to 1.06 and 1.47 when being treated with 25 μM and 50 μM Aftin-4, respectively (Fig 3B). These results were in agreement with previous studies suggesting that Aftin-4 treatment favored the production of amyloid-β 42, which correlated to the neurotoxicity of Alzheimer’s disease [29] (Fig 3C).

In this study, we tested the capability of total isolated proteins for further blot analysis with actin-β and total amyloid-β. Dot blot analysis revealed the presence of actin-β housekeeping protein in 1 μg of total protein (Fig 3D), but could not detect amyloid-β signal. This observation may be due to the low sensitivity of the protein detection by dot blot analysis. The capability of protein detection depends on the amount of the loading protein. Thus, the sensitivity of detection method would depend on the amount of targeted protein. Regardless, we demonstrated that our agarose-laminin scaffold culture system could provide cell population which were able to be extracted for proteins. Besides, the derived proteins could be detected by immunoblotting and ELISA analysis.

### Isolation of RNA from 3D scaffold and subsequent analysis by real-time qRT-PCR

Upon the cell differentiation in the agarose-laminin scaffold, cells were then treated with Aftin-4 for 24 hours to induce amyloidogenic condition in vitro. The RNA of the cell-laden agarose-laminin scaffold was extracted by the TRIzol method and collected from the aqueous phase of TRIzol/chloroform suspension. We found that agarose was also dissolved in the aqueous phase, which resulted in the gelation of the RNA pellet at the end of the extraction procedure. Thus, the agarose removal step was required prior to performing further RNA analysis. To remove the agarose, we used Zymoclean Gel RNA Recovery Kit to recover RNA (Fig 2A). The amount of total RNA after cleaning, determined by Qubit RNA HS was 152 ± 34 ng RNA per 1 x 10^5^ cells (control; n = 4). The integrity of RNA was in a good quality following the cleaning step containing 59% to 75% of structural RNA (determined by the Qubit RNA IQ assay). The size distribution of the RNA fragments was analyzed by an automated on-chip capillary electrophoresis; Bioanalyzer 2100 (S1 Fig). The treatment of Aftin-4 for 24 hours slightly decreased total RNA in a dose-dependent manner but was not statistically significant (*P-value* = 0.44). The results were showed as RNA integrity score (RIN, ranging from score of 1 to 10), which was used to determine the quality of RNA sample. The higher score indicates the higher degree of integrity. The results showed that the RNA extracted by this workflow had RIN score of 6.6 to 7.6 in vehicle control group, 6.1 to 6.8 in 25 μM Aftin-4 treatment group, and 5.6 to 6.6 in 50 μM Aftin-4 treatment group (S1 Fig). The integrity of RNA was lower when treated with higher concentration of Aftin. However, based on manufacturer recommendation, the quality of these derived RNAs exceeded the minimal requirement for both conventional Real-time qRT-PCR and RNA sequencing techniques.

**Fig 4:**
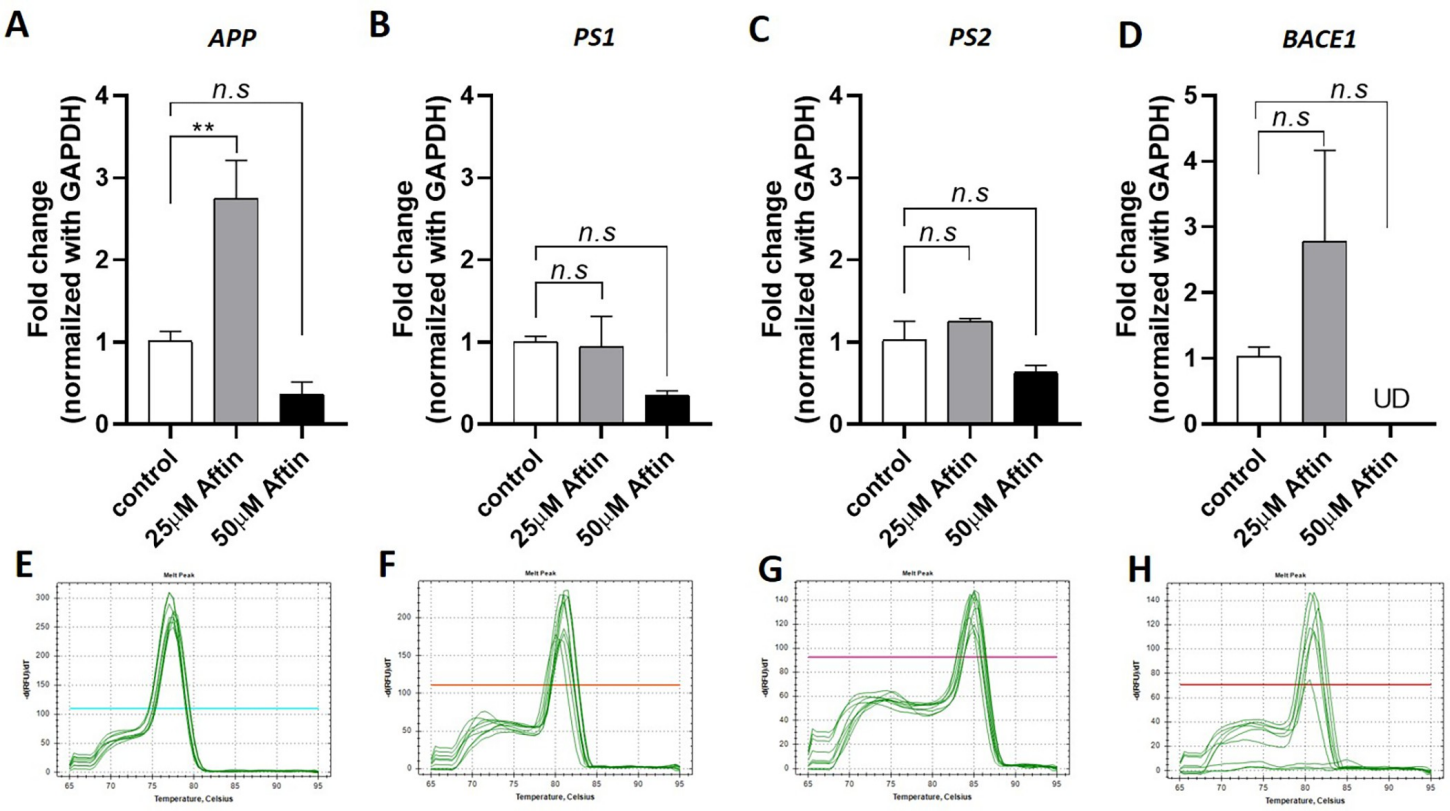
Gene expression analysis A-D) The expression of amyloid-related genes, *APP PS1*, *PS2*, and *BACE1*, respectively upon Aftin-4 treatment for 24 hours. Bar graph represents the mean ± SE (n = 3). **: p<0.01. n.s: not statistically different. E-H) Melt curves analysis of *APP*, *PS1*, *PS2*, and *BACE1*, respectively from all samples.

Here, we showed that the amount of RNA derived from one scaffold or 1 x 10^5^ cells was enough for performing conventional qRT-PCR. However, it needed more than 500 ng of total RNA or about 5 x 10^5^ cells for RNA sequencing, which we did not perform in this study. In this experiment, the derived RNA was proceeded to the reverse transcription prior to qRT-PCR analysis. We measured the expression of the genes in the amyloid-related cascade including *APP*, *BACE1*, *PS1*, and *PS2* following Aftin-4 treatment. We found that 24-hour treatment with 25μM Aftin-4 increased the expression of *APP* to 2.75 folds (*P-value* = 0.01, n = 3), *PS1* to 0.95 folds (*P-value* = 0.97, n = 3), *PS2* to 1.26 folds (*P-value* = 0.49, n = 3), and *BACE1* to 2.78 folds ((*P-value* = 0.07, n = 3). The results represented a uniform melt curve at the temperature 75°C to 85°C. No other peaks were observed, which indicated the efficacy of the qRT-PCR, the cDNA synthesis and RNA extraction (Fig 4E–4H). The upregulation of genes did not alter the amount of total amyloid-β but changed the amyloid beta-42/40 ratio (Figs 3A and 4A–4D). We believed that the increase of amyloid-β 42 and amyloid-β 42/40 ratio might be related to the upregulation of gene(s)-related cascade expression. However, we found that 50μM Aftin-4 treatment resulted in the decrease of gene expressions (Fig 4A–4D). Although the expression of enzyme-encoded genes showed no statistical difference (*P-value* > 0.05), Aftin-4 might exert the production of amyloid-β 42 through its enzymatic activity as described in several previous studies [18, 19]. These results may be due to the instability of RNA at the lethal dose (S1 Fig). Unfortunately, we could not measure the enzyme activity of β-secretase and γ-secretase activity as they require a specific protein extraction method with further optimization to extract an intact enzyme from the agarose-laminin scaffold. Together, we demonstrated that TRIzol extraction is capable to extract RNA from the agarose-laminin scaffold. The trace amount of agarose that was present in the aqueous phase could be removed by using a cleaning column. The derived RNA contained a high percentage of structural RNA for performing gene expression analysis, which allowed us to identify the transcriptomic profiles of amyloid-induced neuronal toxicity.

### Retrieving cell from 3D scaffold and counting cell population by flow cytometry

**Fig 5:**
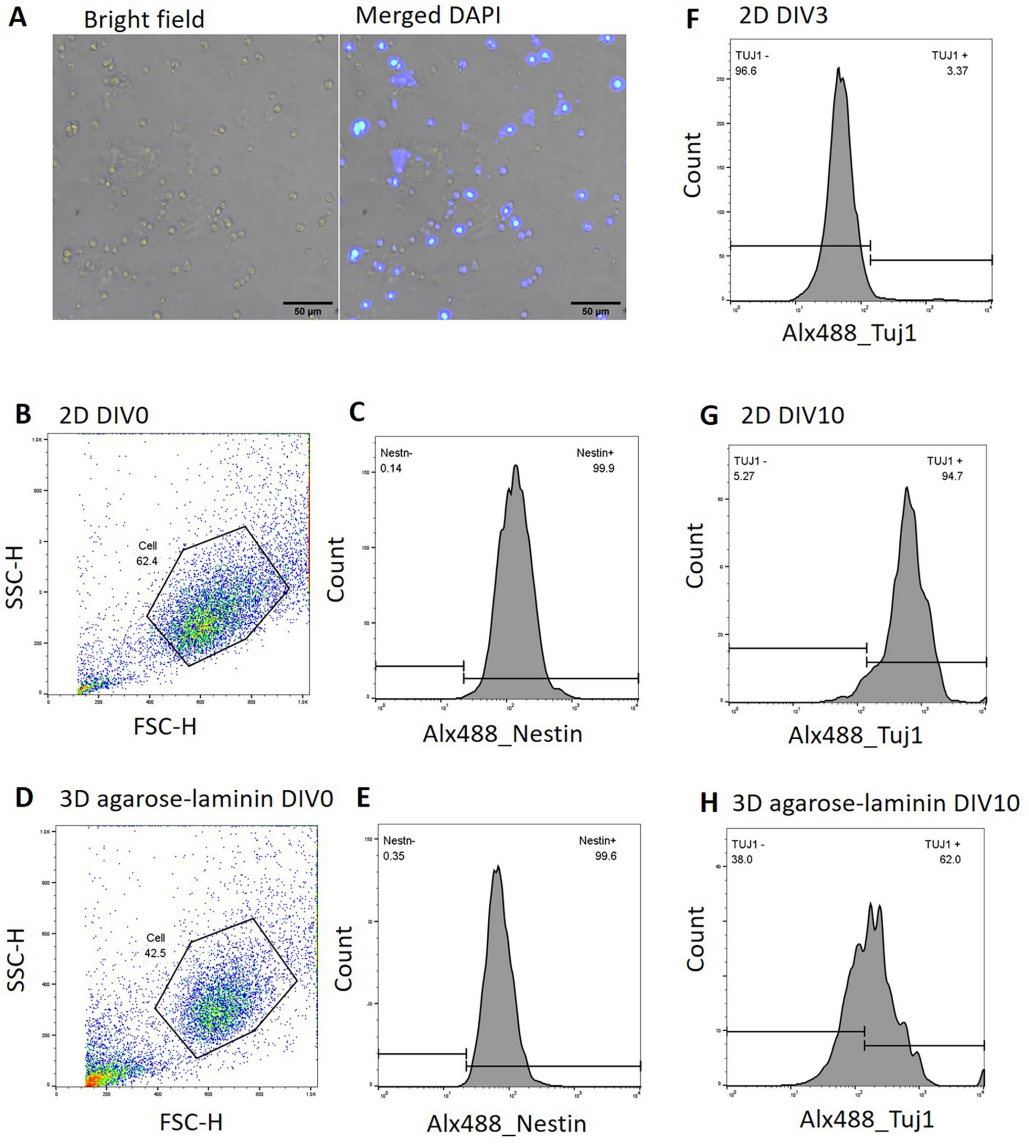
Flow cytometry analysis A) The cell morphology after retrieval and DAPI staining. Scale bars represent 50μm. B) The cellular characteristics; forward scatter (FSC) and side scatter (SSC) of cell derived from 2D culture and D) 3D agarose-laminin scaffold counterpart. C) The histogram of Nestin staining to identify the neural progenitor stage of cells derived from 2D culture and E) agarose-laminin scaffold. F) The histogram of TUJ1 staining to identify the mature neurons derived from 2D culture at DIV3, G) 2D culture at DIV10 and H) 3D agarose-laminin scaffold at DIV10.

One of the limitations in 3D scaffold culture is the detachment of cells out of the scaffold. In this study, we used microwave irradiation to break the agarose structure and retrieved the neurosphere from the agarose-laminin scaffold. Prior to the retrieval step, agarose-laminin scaffolds were submerged in 4% PFA fixative solution for 15 minutes. Call-laden agarose scaffolds were irradiated with intermittent microwave for 5 cycles; 4 seconds on and 4 seconds off. Cells were collected by spin down at 500 x g for 5 minutes. The derived spheroids were dissociated with Trypsin-EDTA enzymatic digestion for 5 minutes and collected by centrifugation (Fig 2B). We showed that cells retrieved from agarose-laminin scaffold was able to be stained with nuclei staining and immunostainings. The cell suspension had positive nuclei staining under a microscope (Fig 5A). To determine the percentage of cell recovery, we performed parallel experiment by preparing cell-laden agarose-laminin scaffold with seeding density of 1 x 10^5^ cells. To avoid the cell proliferation upon cell culture, the cells were immediately retrieved from scaffold after 5 minutes of gelation. The scaffold was fixed with 4% PFA and cell retrieval process was performed as described in material and methods. The cell number of the cell suspension was determined by using cell count kit and the data was acquired by Guava Muse cell analyzer. We found that up to 79.5% of cells could be retrieved by this method (n = 5). We further analyzed the retrieved cells using flow cytometry and compared the cell distribution versus cells derived from 2D culture. The side scatter and forward scatters of both cell cultures in the presence of serum (DIV0) were similar (Fig 5B and 5D). Besides, we found the presence of Nestin-positive cells in both 2D culture and 3D agarose-laminin scaffold culture (99.9% and 99.6%, respectively; Fig 5C and 5E). This result suggested that microwave irradiation did not change the size, the complexity and immunostaining properties of the retrieved cells. In other experiment, we proceeded the cell culture in the scaffolds prior to TUJ1 staining. The results showed the presence of TUJ1-positive cells of 3.37% at DIV3 and increased to 94.7% at DIV10 in 2D culture (Fig 5F and 5G), while the cells retrieved from 3D agarose-laminin scaffold showed 62% of TUJ1-positive cells (Fig 5H). We found the evidence of cell cycle shifting from G2/M phase to G0/G1 phase after being cultured in differentiation medium for 24 hours (S2B and S2D Fig). In addition, the differentiation of cells in 3D agarose-laminin scaffold altered the size and the complexity of the cells, determined by forward- and side-scatter of flow cytometry (S2A and S2C Fig). Together, these observations indicated that the cells were capable to differentiate toward matured TUJ1-positive neurons in agarose-laminin scaffold. The morphology and the complexity of cells under in agarose-laminin scaffold was differed from 2D culture.

We also attempted to perform a fluorescence immunostaining of 3D scaffold prior to image analysis. We used double staining calcein-AM and propidium iodide to determine cell viability. We found that calcein-AM was not stable in agarose scaffold, as agarose has autofluorescence at the wavelength of 400 to 500 nm, which is a similar emission wavelength of calcein-AM. However, DAPI and propidium iodide were suitable for fluorescence staining of agarose-laminin scaffold. We did not perform immunostaining in the gel as it may require the optimization of antibody concentration and the staining times, which might not be enough for penetration into the gel and cells. To overcome this limitation, using fluorescence labeling cells in agarose-laminin would be a good option to skip the post-immunostaining step. Although this study had failed to perform immunostaining of 3D culture, we showed that this workflow was suitable for cell retrieval. The retrieved cells still had intact nuclei and were suitable for flow cytometry analysis as similar as 2D conventional culture. In addition, cells cultured in agarose-laminin showed low variation of spheroid size and were within a good cell cycle stage. We expected that our workflow could be implemented to identify the cellular and molecular responses relating to amyloid-β-induced neural toxicity. Together, this workflow provides a potential tool to study the mechanisms related to the amyloidogenic cascade in neurodegenerative disorders such as Alzheimer’s disease.

## Discussion

In general, traditional cell culture has been established in 2D platforms. Cells were plated onto a plastic surface and spread to cover the surface area. This method is popularly used as it is easy to manipulate and has good cost-efficiency. Due to its well-known and popularity, several methods to analyze cellular and molecular mechanisms are well optimized and validated, such as DNA-, RNA-, protein-extraction, transcriptomic and proteomic analysis, as well as cell imaging and flow cytometry. Although 2D cell culture has several validated support protocols to study cellular and molecular mechanisms, it does not recapitulate the actual cell developmental environment and thus may not be pathological relevant. On the other hand, in 3D culture system, where cells could grow mimicking actual environment with or without scaffold, can provide an actual environment in the disease context, and thus has pathological importance than the previous 2D culture platform [35]. A previous study showed that cell culture in a 3D environment had higher viability and growth rate (ref). We also observed similar pattern in our cell culture, which we found the detachment of neurons in 2D culture after 10 days, However, cell-laden agarose-laminin culture was still intact and remained viable in culture up to 21 days. Of this advantage, the 3D culture will be suitable for such prolonged experiment. More importantly, cells in 3D culture could secrete higher amount of protein compared with the traditional 2D culture [38]. This would facilitate the study of secretory protein in cell culture system. Since cell culture was a tool generated for studying disease models, 3D culture was proposed in multiple platforms such as with and without scaffold [33, 34]. Among these, free-floating culture is popular and easy to generate but the size of spheroids was varied depending on the seeding density. Therefore, several researchers have developed a 3D culture which allows to specify the seeding density and spheroid size [39, 40]. Some of these methods required a 3D printer to make a mold. Besides these platforms, an alternative method to control the size of a spheroid is to grow cells inside a synthetic scaffold or matrix. With this method, the researcher was capable to add extracellular matrix (ECM) into the culture, which generates an environmental-like structure that supports the cell culture and enables us to study cell-ECM interaction.

Due to the advantage of 3D culture over 2D conventional culture, several disease models are being developed in 3D platforms, including Alzheimer’s disease. The pathological hallmark of Alzheimer’s is believed to begin with the amyloid-β accumulation. The imbalance of amyloidogenic pathway leads to the increase of amyloid-β 42 over other isoforms. Previous studies have shown that the increase of amyloid-β 42 deposit enhances its aggregation and becomes more neurotoxic form [41, 42]. However, the abundance of amyloid-β 42 alone does not correlate with the pathological signs. Instead, the shift of amyloid-β 42/40 ratio is more relevant to the disease [29, 43, 44]. Due to the lack of an appropriate model, the mechanism involving amyloid-induced neuronal toxicity is still underdetermined. Some of previous studies used the treatment of high concentration of exogenous synthetic amyloid-β 42 and assessed the cellular and molecular response toward amyloid toxicity. However, the disease progression is initiated by the gradual accumulation of toxic protein. Thus, the treatment of exogenous synthetic protein is pathological irrelevant in the early stage of the disease. Therefore, several studies used cell line carrying inherited mutation gene(s), recapitulating the development of FAD. However, FAD is only accounted for 10% of total Alzheimer’s cases, while 90% are sporadic and do not carry mutation gene(s). For this reason, small-molecule-induced amyloidogenic condition becomes a suitable model for studying the progression of Alzheimer’s disease. This study illustrated the preparation of cell-laden agarose-laminin scaffold 3D culture. To demonstrate the cellular and molecular analysis of this 3D scaffold, we created the amyloidogenic condition using amyloid-42 inducer small molecule. This small molecule was previously used to generate Alzheimer’s like conditions both in vitro and in vivo [18–20]. In agreement with several previous studies, we demonstrated that the Aftin-4 small molecule selectively induced amyloid-β 42 deposit, which is the pathological isoform in Alzheimer’s brain. Here, we showed the workflow beginning from 3D culture preparation and subsequent RNA and protein extraction, and cell retrieval from the scaffold for further analysis. We showed that RNA and protein could be extracted from the agarose-laminin scaffold by using TRIzol assay. The amount of total RNA per 10^5^ cells was 152± 34 ng which contained about 59% to 75% structural RNA or RIN score of 5.6 to 7.6. The quality and quantity of these RNAs were good enough for traditional one-step and 2-step qPCR. According to the manufacturer, the RNA which contained more than 60% structural RNA or RIN score above 6.0 can be used to perform RNA sequencing with a good result. This indicates that RNA-derived from this 3D culture could be further utilized in transcriptomic RNA sequencing. However, we did not go further with RNA sequencing in this study. Besides, we showed that we could isolate the total protein of 2.81± 0.8 μg from 10^5^ cells in a 3D scaffold. We then evaluated the amount of total amyloid-β (40 plus 42), about 60.42± 12.96 pg/ml present in the 1 μg total protein, and found that the induction of amyloid-β 42 by Aftin-4 gave the concurrent results similar as previous studies [19, 20]. The treatment of Aftin-4 for 24 hours altered the amyloid-β 42/40 ratio in a dose-dependent manner. This result suggested that protein derived from 3D scaffold was intact and capable to perform an ELISA assay. More importantly, the isolated protein from the scaffold culture system can be further utilized in downstream conventional protein analysis, such as protein blot analysis etc. Although the total protein isolated from 1x10^5^ cells were considerably low for a western blot analysis, dot blot analysis was doable. Pooling samples is recommended if western blot analysis is preferred. Amyloid-β is a secretory protein that is normally dissolved in cells culture medium when growing cells in a 2D platform. In this present study, all cells were laden inside the agarose hydrogel scaffold. The secreted proteins were dissolved in both scaffolds and also diffused into the cell culture medium as the medium moves freely between two phases. In comparison with the qPCR analysis of amyloid-related genes, we found the increase of *APP* gene and the increase of amyloid-β 42/40 ratio at the sublethal dose; 25μM Aftin-4 only, but not at the dose of 50 μM Aftin-4. This may be caused by the regulation of enzyme activity level to produce amyloid-β by Aftin-4 small molecule and the instability of mRNA after exposure to the higher dose of Aftin-4 for 24 hours as shown by lower RIN score in 50 μM Aftin-4 treatment group (S1 Fig). Nevertheless, this study demonstrated that the extraction of RNA and protein from agarose-laminin scaffold could be done with the traditional TRIzol method which both RNA and protein were extracted from a single sample. Thus, it could reduce the variation from batch to batch analysis. We found the trace amount of agarose dissolved in the upper aqueous phase of TRIzol extraction and could be removed by column cleaning. The treatment of Aftin-4 resulted in cytotoxicity, which affected the amount of total RNA (*P-value* = 0.44) and protein (*P-value* = 0.42) but was not statistically different in this study.

In addition, we first demonstrated the cell retrieval from the agarose-laminin scaffold. The derived cells were then used for flow cytometry analysis. We found that the distribution of side scatter and forward scatter of cells derived from 3D agarose scaffold looked similar to a cell derived from a 3D culture without a scaffold at the early period of the culture. However, the differentiated neurons in 3D culture had higher complexity and variation in size distribution (S2A and S2C Fig), suggesting that the morphology of cells culture in 3D culture behaves different from 2D culture. This study demonstrated that the intermittent microwave irradiation method was suitable for cell retrieval from agarose-laminin scaffold with 79.5% recovery and suitable for immunostaining and nuclei staining. The propidium iodide cell cycle analysis revealed that cell-laden agarose-laminin exhibited cell differentiation after being cultured in differentiation medium for 24 hours (or DIV3; S2D Fig). This observation suggested that the retrieval method had a minimal change to cell integrity. Upon culturing in differentiation medium until DIV10, the retrieved cells were then stained with TUJ1 marker, which is normally expressed at this time point.

A previous study showed that cells expressed higher *APP* gene expression as culture was prolonged [20]. Thus, a longer cell culture could produce more amyloid-β secretion and resulted in the aggregation of the toxic isoform. In addition, a study using FAD ReN cells showed that amyloid-β aggregation was initiated around 6 weeks of cell culture while phosphorylated-tau protein was presented around 10 weeks in a 3D platform [24]. This 3D culture method could be maintained for a longer period as the morphology of the cells looked healthy inside the scaffold on day 21 of culture. These results proved that 3D culture could be an excellent model for studying Alzheimer’s disease. However, this workflow finished the experiment within a week and did not further investigate another pathological hallmark of Alzheimer’s disease. So far, this protocol has a limitation on 3D-immunostaining and 3D-imaging. The 3D image should be taken in z-stack mode of a high-performance microscope. In this study, we tried to dissect 3D agarose/laminin gel cell culture to a thin layer, which would allow us to stain and observe under a conventional fluorescence microscope. However, the embedded cells in 0.75% agarose gel was soft and unable to dissect into a thin slide. The method of clearing agarose gel or embedded cell-gel scaffold should be further optimized to overcome the 3D-immunostaining and 3D-imaging limitation. Nevertheless, we showed that the staining of a nuclei-specific dye such as DAPI and propidium iodide and immunostaining of retrieved cells were doable, which indicated that cells were still intact after the cell retrieval procedure. We believed that the limitation of 3D-imaging could be overcome by using a fluorescence reporter cell line which would skip the post-labeling steps. However, the optimization should be further investigated. Taken together, this study demonstrated the workflow of 3D culture in the agarose-laminin scaffold. This method is feasible, simple, and cost-efficiency. The derived RNA protein and cells from this 3D culture allow the user to further investigate the cellular and molecular mechanisms of cells. Here, we also showed the induction of amyloidogenic condition by using amyloid-β 42 inducer. The treatment of 25μM Aftin-4 for 24 hours increased *APP* expression and amyloid-β 42/40 ratio in neuron-like cell line; SH-SY5Y without carrying AD-related genes. This 3D culture could be used as a model of sporadic Alzheimer’s (SAD) which patients do not inherit AD-risk genes. In addition, the small molecule could induce the accumulation of amyloid-β and sustained the amyloid-β deposit inside the scaffold. Thus, this model could recapitulate the early stage of the disease. This 3D scaffold culture is simple and convenient. Most of the reagents are regularly used in cell culture and molecular laboratories. It can help researchers determine the molecular mechanism underlying the amyloidogenic cascade, as well as use this 3D neuronal scaffold culture for cellular and molecular analysis. To take a step forward toward a high-throughput assay, the combination of 3D agarose-laminin scaffold with a thermal controlled 3D printer would have been interesting to explore a high-throughput drug screening. Besides, the evaluation of other pathological hallmarks of Alzheimer’s disease such as amyloid-β oligomers, neurofibril tangle, and mitochondrial dysfunction have not been done in this study and should be further investigated.

## Supporting information

S1 Fig RNA fraction. The analysis of RNA fraction from 3 different conditions; vehicle control, 25μM Aftin-4 and 50μM Aftin-4 using Agilent Bioanalyzer 2100 instrument and Agilent RNA Pico Kit (n = 4).
(TIF)

S2 Fig Cell cycle analysis using propidium iodide dye. A) The cellular characteristics; forward scatter (FSC) and side scatter (SSC) and B) propidium iodide staining of cell derived from DIV0 C) The cellular characteristics; forward scatter (FSC) and side scatter (SSC) and D) propidium iodide staining of cell derived from3D agarose-laminin scaffold DIV3.
(JPG)
