# Correction: An in vitro workflow of neuron-laden agarose-laminin hydrogel for studying small molecule-induced amyloidogenic condition

**DOI:** 10.1371/journal.pone.0315229

**Published:** 2024-12-04

**Authors:** The PLOS ONE Staff

The fifth author’s initials appear incorrectly in the citation. The correct citation is: Namchaiw P, Bunreangsri P, Eiamcharoen P, Eiamboonsert S, Poo-arporn RP (2022) An in vitro workflow of neuron-laden agarose-laminin hydrogel for studying small molecule-induced amyloidogenic condition. PLOS ONE 17(8): e0273458. https://doi.org/10.1371/journal.pone.0273458.

There is an error in affiliation 3 for author Piyaporn Eiamcharoen. The correct affiliation 3 is: Department of Pathology, Faculty of Veterinary Medicine, Kasetsart University, Chatuchak, Bangkok, Thailand.

The publisher apologizes for the errors.
