# Supplementary figures and images for: An in vitro workflow of neuron-laden agarose-laminin hydrogel for studying small molecule-induced amyloidogenic condition
Source: PLoS One. 2022 Aug 26;17(8):e0273458. doi: 10.1371/journal.pone.0273458 (PMC9416999; doi:10.1371/journal.pone.0273458)

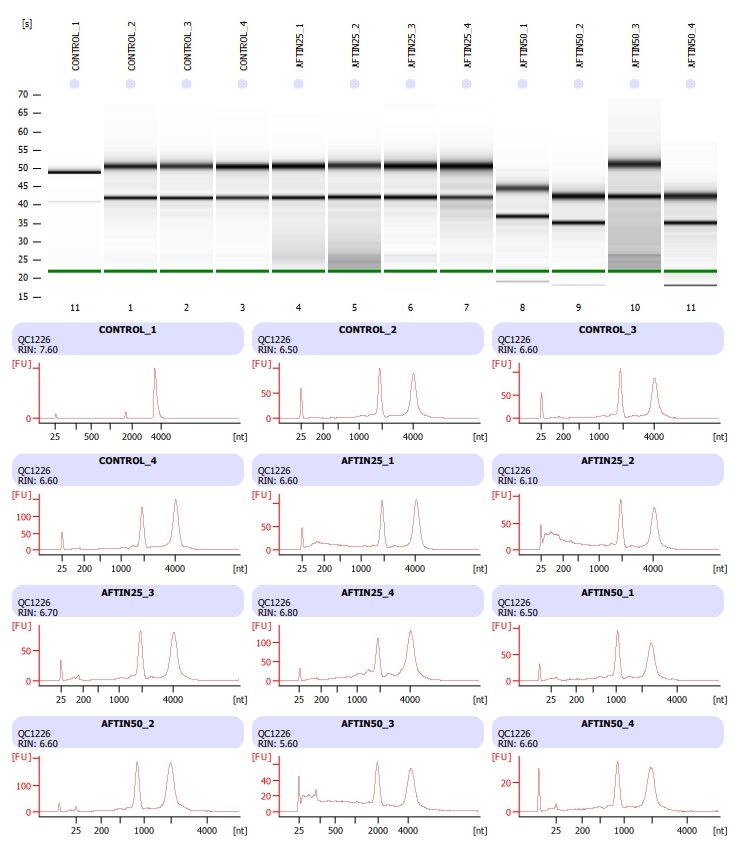

Supplement: S1 Fig — The analysis of RNA fraction from 3 different conditions; vehicle control, 25μM Aftin-4 and 50μM Aftin-4 using Agilent Bioanalyzer 2100 instrument and Agilent RNA Pico Kit (n = 4). (TIF) [file pone.0273458.s001.tif]

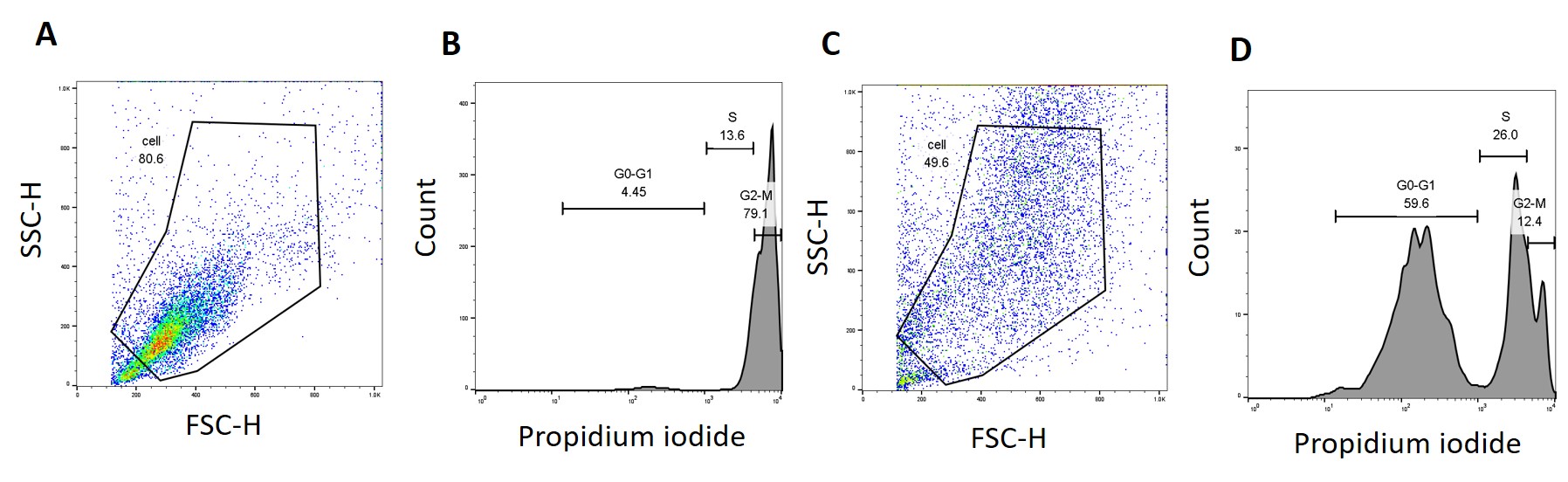

Supplement: S2 Fig — A) The cellular characteristics; forward scatter (FSC) and side scatter (SSC) and B) propidium iodide staining of cell derived from DIV0 C) The cellular characteristics; forward scatter (FSC) and side scatter (SSC) and D) propidium iodide staining of cell derived from3D agarose-laminin scaffold DIV3. (JPG) [file pone.0273458.s002.jpg]
